# Bridging Innovation and Clinical Insights: Reflections on Healthcare Research and Emergency Medicine

**DOI:** 10.51894/001c.124544

**Published:** 2024-09-10

**Authors:** Francis O. Akenami, Rana Ismail, Andrea Amalfitano

**Affiliations:** 1 College of Osteopathic Medicine- Statewide Campus System, East Lansing, MI 48824 Michigan State University

**Keywords:** emergency medicine, gastroenterology, otolaryngology, Quality Improvement, Orthopedic and surgical innovations, total knee arthroplasty, Frontal Sinus Balloon Sinuplasty, Current Pharmacologic Options

In the face of evolving healthcare demands, Volume 9, Issue 3 of the *Spartan Medical Research Journal* brings together an array of studies highlighting the blend of innovative techniques, evidence-based practices, and clinical insight across multiple medical disciplines. From emergency medicine to gastroenterology and otolaryngology, the seven articles in this issue offer valuable contributions that enhance both patient care and operational efficiency.

One of the highlights is the article by Singh et al, *Contemporary Trends in Frontal Sinus Balloon Sinuplasty*, which explores the utilization of balloon sinuplasty (BSP) in treating chronic rhinosinusitis.^1^ Their findings revealed that, despite the increasing use of BSP in both hospital and office settings, there remains a high rate of complications, particularly in the frontal sinuses. The research underscores the need for further studies to refine these techniques and minimize complications, particularly in community-based hospitals where this procedure is performed more frequently.

Another important addition to this issue is the article by Sebastian et al., *Current Pharmacologic Options and Emerging Therapeutic Approaches for the Management of Ulcerative Colitis*.^2^ Their comprehensive review highlights new therapeutic agents that hold promise for patients with ulcerative colitis (UC), particularly those unresponsive to traditional therapies such as 5-aminosalicylic acid (5-ASA) and corticosteroids. Novel biologics, anti-integrin molecules, and Janus kinase (JAK) inhibitors offer new avenues for treatment, with emerging evidence suggesting that they may reduce long-term reliance on steroids, thus minimizing associated side effects. This review provides clinicians with up-to-date treatment options, equipping them to better manage UC in their practices.

In keeping with the focus on practical advancements in patient care, Ghiardi et al.’s *Resident-Led Quality Improvement Project in a Community-Based Hospital Emergency Department* illustrates how small operational changes can yield significant clinical benefits.^3^ The introduction of a simple suture cart in the emergency department not only reduced laceration repair times by over 30 minutes but also demonstrated the value of quality improvement projects in a resource-constrained environment. The success of this project serves as a testament to how effective, low-cost interventions can improve patient throughput without overburdening staff.

The importance of improving prescribing practices is underscored by Nedzlek et al., who examined corticosteroid usage in treating chronic obstructive pulmonary disease (COPD) exacerbations in the emergency department.^4^ Their findings reveal that evidence-based adjustments in corticosteroid dosages significantly reduced patient length of stay, reinforcing the critical role that proper dosing plays in optimizing patient outcomes in emergency settings.

Singh et al.’s article, *Clinical Predictors of Hospital-Acquired Bloodstream Infections: A Healthcare System Analysis*, sheds light on one of the most serious complications in hospitalized patients.^5^ By identifying key predictors such as male sex, fever, and lymphopenia, this study offers a predictive model that could help healthcare providers better assess the risk of hospital-acquired bloodstream infections (HABSI), thereby improving antibiotic stewardship and patient management.

Orthopedic and surgical innovations also take center stage in this issue. Fry et al. investigated the *Influence of Porosities of 3D Printed Titanium Implants,*^6^ finding that increased porosity improves tendon integration in animal models. This research holds the potential to revolutionize orthopedic surgeries, particularly in cases of tendon repair, where the need for durable, high-strength implants is critical.

Finally, Zondervan et al. explored the impact of postoperative positioning in total knee arthroplasty patients.^7^ Their study demonstrates that patients who adopt a supine sleeping position experience significantly better knee extension post-surgery, suggesting a simple yet effective change in post-surgical care protocols that could reduce complications and improve recovery outcomes.

The articles presented in this issue illustrate the breadth of research and innovation that is driving clinical excellence across a variety of fields. Whether through novel pharmacologic approaches, advanced surgical techniques, or practical quality improvement initiatives, each study contributes to the ongoing effort to enhance patient care, optimize operations, and ultimately improve healthcare outcomes. We hope that these contributions inspire further advancements in both research and practice.
